# Roles of Rad51 paralogs for promoting homologous recombination in *Leishmania infantum*

**DOI:** 10.1093/nar/gkv118

**Published:** 2015-02-24

**Authors:** Marie-Michelle Genois, Marie Plourde, Chantal Éthier, Gaétan Roy, Guy G. Poirier, Marc Ouellette, Jean-Yves Masson

**Affiliations:** 1 Genome Stability Laboratory, CHU de Québec Research Center, HDQ Pavilion, Oncology Axis, 9 McMahon, Québec City, QC G1R 2J6, Canada; 2 Department of Molecular Biology, Medical Biochemistry and Pathology, Laval University, Québec City, QC G1V 0A6, Canada; 3 Centre de Recherche en Infectiologie, CHUL, 2705 boul. Laurier, Quebec, Quebec G1V 4G2, Canada; 4 CHU de Québec Research Center, CHUL Pavilion, Oncology Axis, 2705 boul. Laurier, Quebec city, Quebec, G1V 4G2, Canada

## Abstract

To achieve drug resistance *Leishmania* parasite alters gene copy number by using its repeated sequences widely distributed through the genome. Even though homologous recombination (HR) is ascribed to maintain genome stability, this eukaryote exploits this potent mechanism driven by the Rad51 recombinase to form beneficial extrachromosomal circular amplicons. Here, we provide insights on the formation of these circular amplicons by analyzing the functions of the Rad51 paralogs. We purified three *Leishmania infantum* Rad51 paralogs homologs (*Li*Rad51-3, *Li*Rad51-4 and *Li*Rad51-6) all of which directly interact with *Li*Rad51. *Li*Rad51-3, *Li*Rad51-4 and *Li*Rad51-6 show differences in DNA binding and annealing capacities. Moreover, it is also noteworthy that *Li*Rad51-3 and *Li*Rad51-4 are able to stimulate Rad51-mediated D-loop formation. In addition, we succeed to inactivate the *LiRad51*-*4* gene and report a decrease of circular amplicons in this mutant. The *LiRad51*-*3* gene was found to be essential for cell viability. Thus, we propose that the *Li*Rad51 paralogs play crucial functions in extrachromosomal circular DNA amplification to circumvent drug actions and preserve survival.

## INTRODUCTION

In response to various types of DNA damage, every organism relies on DNA repair pathways to preserve genomic integrity. To be accurate, the error-free double-strand break (DSB) repair named homologous recombination (HR) can use sister chromatids, as a repair template, for homology search and strand invasion (1). A key player of homologous recombination is the eukaryotic RAD51 recombinase (RecA homolog in *Escherichia coli*). Following the resection step carried out by MRE11-RAD50-NBS1, CtIP, EXO1, BLM and DNA2, replication protein A (RPA) coats the single-strand DNA of the 3′-resected DNA tails (2). Mediators, such as BRCA2, assist RAD51 to displace the RPA to allow RAD51 to polymerize as nucleoprotein filaments on the resected DNA (3,4). Then, RAD51 invades the homologous sequence to form joint molecules referred as displacement-loop (D-loop). This process is facilitated by the recruitment of many proteins such as RAD52, BRCA2, PALB2 and also the five mammalian RAD51 paralogs (RAD51B, RAD51C, RAD51D, XRCC2, XRCC3) (5–10). These proteins which probably derived from RAD51 duplication events (11), bear 20–30% identity at amino acid level with RAD51 and with each other. Conserved sequences are found in the walker A and walker B domains, which contain nucleotide-binding motifs conferring ATP binding and weak hydrolysis activity (12–14). The RAD51 paralogs function in different complexes and sub-complexes. RAD51C is present in two main complexes termed BCDX2 (formed of RAD51B-RAD51C-RAD51D-XRCC2) and CX3 (RAD51C-XRCC3) (15). Sub-complexes RAD51C-RAD51B, RAD51C-RAD51D, RAD51C-XRCC3 and RAD51C-RAD51D-XRCC2 have been also detected. RAD51 and RAD51 paralogs complexes interact together as judged by yeast three-hybrid experiments and coimmunoprecipitation assays (15–18). Consistent with this result, Rodrigue *et al*. demonstrated *in vivo* interaction in mammalian cells between RAD51 and the central core factor, RAD51C (19).

Multiple evidence highlight the importance of RAD51 paralogs in DNA damage repair and HR acting as cofactors for RAD51 filament formation (20–27). Mutants in each individual RAD51 paralogs display chromosome instability, growth retardation and decrease of RAD51 foci formation after DNA damage (10,28–34). Recently, it has been suggested that RAD51 paralogs are essential to drive recombinational repair by acting in early and late stages of HR, but individual functions for these factors remain difficult to assess (35,36). Interestingly, four paralogs (RAD51B, RAD51C, RAD51D, XRCC3) contain at their N-terminus a conserved helix-hairpin-helix motif require for non-sequence specific DNA binding (18). Furthermore, successful homogeneous purification of both human BCDX2 and CX3 protein complexes allowed DNA binding characterization and visualization by electron microscopy. As expected, BCDX2 and CX3 bound preferentially single-stranded DNA (ssDNA) and 3′-tail and 5′-tail DNA substrates (15,37). Electron micrographs of BCDX2 and CX3 revealed a strong binding for the junction of the replication fork structure and the intersection of the four duplex arms in the Holliday junction (38). The complexes might recognize the ssDNA at the junction. It has also been documented that RAD51C stimulates RAD51-mediated strand exchange and is able to catalyze by itself homologous pairing for D-loop formation (18,39).

Homologous recombination is also evolutionary conserved in Trypanosomatidae. Specifically, in *Trypanosoma brucei*, HR is important for antigenic variation while in *Leishmania*, it catalyzes DNA amplification through rearrangements of the whole genome as a strategy to overcome drug resistance (40,41). The parasite *Rad51* gene bears 60–80% sequence identity with human *RAD51* (42,43). *RAD51* knockout is lethal as demonstrated in mammalian and chicken cell lines (44–46) but successful generation of *Rad51* null mutant in *T. brucei* leads to viable progeny. The mutant exhibits a sensitivity to DNA damaging agents (methylmethane sulfonate (MMS) and 3-aminobenzamide) and a partial impairment of VSG (Variant Surface Glycoprotein) switching showing that Rad51 has a role in antigenic variation through HR (47). The deletion of the *Rad51* gene in *Leishmania infantum* was also achievable and provided insight on the mode of action of HR protein involved in resistance by gene amplification (48). In fact, *Li*Rad51 is important for the formation of circular amplicons through repeated sequences but is not essential (48). This observation suggests the presence of other players involved in this mechanism. As the two main recombination mediators RAD52 and PALB2 are undetectable by homology in *Trypanosoma brucei, Trypanosoma cruzi and Leishmania major* (49), the search for RAD51-related proteins indicated that *Trypanosoma* species possess four Rad51 paralogs (Rad51-3, Rad51-4, Rad51-5 and Rad51-6) and only three in *Leishmania* (Rad51-3, Rad51-4 and Rad51-6). Protein alignments with the human proteins indicate that Rad51-3 is closest to RAD51C, Rad51-4 to XRCC2, Rad51-5 to XRCC3 and Rad51-6 to RAD51D (49). Functional analysis is most extensive for *T.brucei*, where each Rad51 paralogs mutant has been examined. The contribution of individual paralog provided evidence for distinct functions, for instance, only Rad51-3 is involved in VSG switching. Likewise, each null mutant displays sensitivity to phleomycin and MMS, gene-targeting defects and reduction of Rad51 foci formation at DNA damage sites (43,50). The interactions among *T.brucei* Rad51 paralogs 3,4 and 6 indicate either a stable trimer or dimeric complexes (50). The fourth Rad51 paralogs, Rad51-5, that is not identified in the *Leishmania* pathogen, failed to interact with the other paralogs.

To date, the biochemical characterization of Trypanosomatidae Rad51 paralogs has not been studied and no report unveils *in vivo* or *in vitro* interaction with Rad51. Here, we present the first report showing purification of *Li*Rad51 paralogs and its interaction with *Li*Rad51. We show evidence that the *Li*Rad51 paralogs promote HR through their capacity to bind and anneal DNA and also stimulate D-loop formation. Remarkably, we also provide insights on their role in gene amplification, a stochastic mechanism to respond to drug and adapt to a changing environment.

## MATERIALS AND METHODS

### Nucleic acids

*L. infantum* Rad51 paralogs genes were obtained by polymerase chain reaction (PCR) amplification from intronless *Leishmania* genomic DNA. PCR fragments were amplified with the combination of primers JYM2212 and JYM2213 for *Li*Rad51-3 (NCBI reference E9AHR7), JYM2216 and JYM2217 for *Li*Rad51-4 (NCBI reference A4HUT5), and JYM2218 and JYM2219 for *Li*Rad51-6 (NCBI reference A4I499) (Supplementary Table S1). The genes were cloned in a modified pFASTBAC1 plasmid (Invitrogen) encoding GST and His tags (51). Similarly, the genes were also cloned in pFASTBAC1 plasmid (Invitrogen) where distinct tags (Myc, V5 or HA tag) were respectively added at the C-terminus of each protein. *Li*Rad51-3-Myc was generated with primers JYM2228 and JYM2229, *Li*Rad51-4-V5 was produced with primers JYM2230 and JYM2231 and finally *Li*Rad51-6-HA was obtained by primers JYM2226 and JYM2227 (Supplementary Table S1). All constructs were confirmed by DNA sequencing.

### Generation of the *LiRad51*-*3* and *LiRad51*-*4* null mutant

Knockout of the first allele of *LiRad51*-*3* (*LinJ33_V3.2620*) was generated by the substitution of the entire open reading frame (ORF) by a hygromycin phosphotransferase cassette flanked by 5′ and 3′ regions of the gene. The upstream *LiRad51*-*3* region was PCR amplified with primers JYM2129 and JYM2120 (Supplementary Table S1) and the downstream region was obtained with primers combination JYM2122 and JYM2123 (Supplementary Table S1) from *L. infantum* genomic DNA. Targeting flanks were ligated to the *HYG* marker gene amplified by PCR with primers JYM1791 and JYM2121 (Supplementary Table S1) as previously reported (52). We obtained the *LiRad51*-*3* null mutant only in condition where the episomal copy of the gene *LinJ33_V3.2620* was present. *LiRad51*-*3* was cloned in pSP72α*PURO*α using primers JYM2772 and JYM2773 and the construction was transfected in *L. infantum LiRad51*-*3^+/−^*. The inactivation of the second allele of *LiRad51*-*3* was performed by an insertional inactivation strategy where the neomycin phosphotransferase cassette was fused with the 200 pb of the end of the gene to target the remaining allele. The *NEO* marker gene was PCR amplified using the primers JYM1797 and JYM2126 while the 5′ and 3′ flanking regions were obtained using primers JYM2124 and JYM2391 (upstream region) and primers JYM2127 and JYM2128 (downstream region) (Supplementary Figure S7 and Supplementary Table S1). Integration of *HYG* and *NEO* cassettes was confirmed by PCR using primers pairs a+b, a+c and a+d where “a” is JYM3083, “b” is JYM3084, “c” is JYM3085 and “d” is JYM2126 and the primers MOUC2 et MOUD2 for episomal PURO cassette (Supplementary Table S1).

The first round of *LiRad51*-*4* (*LinJ11_V3.0230*) inactivation was achieved by a neomycin phosphotransferase cassette fused with the 200 pb of the end of the gene. The *NEO* marker gene was PCR amplified using the primers JYM1797 and JYM2136 while the 5′ and 3′ flanking regions were obtained with primers JYM2134 and JYM2392 (upstream region) and primers JYM2137 and JYM2138 (downstream region) (Supplementary Table S1). The second round of inactivation was performed by the substitution of the entire ORF by a hygromycin phosphotransferase cassette amplified using primers JYM1791 and JYM2131 flanked by 5′ and 3′ regions of the gene generated by JYM2129 and JYM2130 (upstream region) and primers JYM2132 and JYM2133 (downstream region) (Figure 6 and Supplementary Table S1). For episomal complementation of *LinJ11_V3.0230, LiRad51*-*4* gene was cloned in pSP72α*PURO*α using primers JYM2774 and JYM2775 and the construction was transfected in *L. infantum LiRad51*-*4^−/−^* (Supplementary Table S1). Integration of *HYG* and *NEO* cassettes was confirmed by PCR using primers pairs a+b, a+c, a+d 1+6 and A+B where “a” is JYM2834, “b” is JYM2832, “c” is JYM2136, “d” is JYM3085, “1” is JYM2834, “6” is JYM2138, “A” is JYM2774 and “B” is JYM2775. (Supplementary Table S1). As previously published (53) selection of recombinants and transfectants were obtained as following: 5 μg of both final targeting linear inactivation cassettes (*NEO* or *HYG*) or 20 μg of plasmid vector for episomal complementation were transfected into promastigotes by electroporation. Depending on the marker used, selection was achieved in the presence of 600 μg/ml hygromycin B, 40 μg/ml G418 (Geneticin, Gibco-BRL) and 100 μg/ml puromycin.

**Figure 1.**
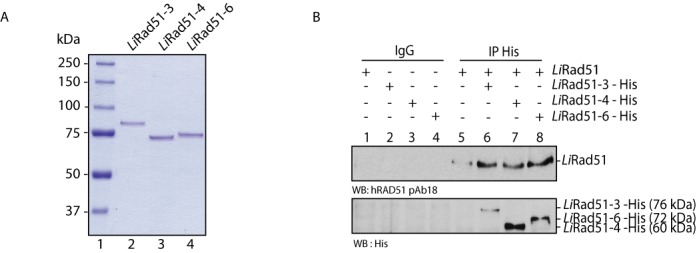
Purification and interactions of *Li*Rad51 paralogs, **(A)** SDS-PAGE of purified *Li*Rad51 paralogs. Purified proteins (300 ng) were loaded on a 8% SDS-PAGE, and stained with coomassie blue. Lane 1, molecular weight markers (Bio-Rad); lane 2, purified *Li*Rad51-3; lane 3, purified *Li*Rad51-4; lane 4, purified *Li*Rad51-6. **(B)** Interactions between purified *Li*Rad51 and paralogs. *Li*Rad51 and *Li*Rad51-3 or *Li*Rad51-4 or *Li*Rad51-6 were incubated together and co-immunoprecipitated using either IgG (lanes 1–4) or His (lanes 5–8) antibodies. Blots were revealed with the indicated antibodies.

**Figure 2.**
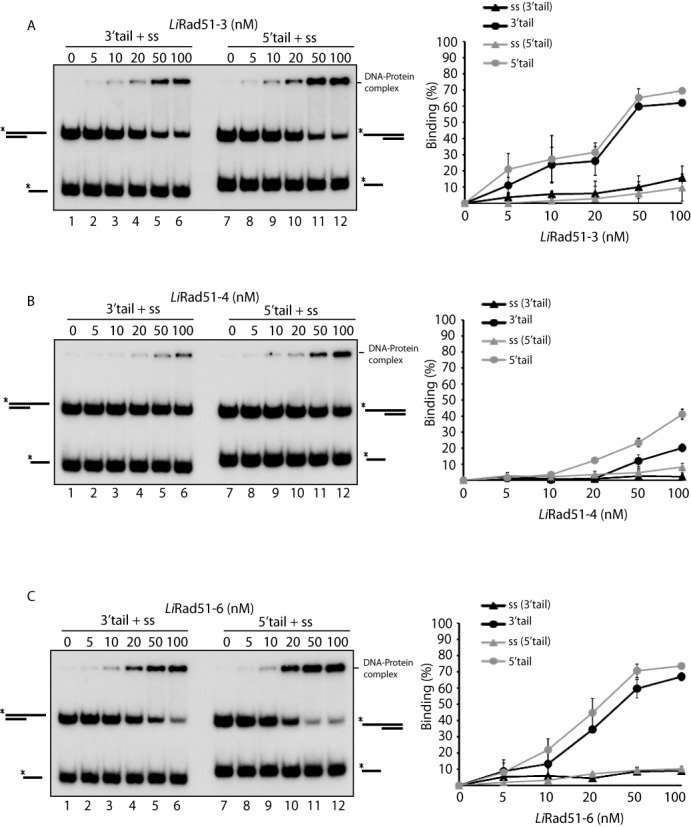
DNA binding activity of purified *Li*Rad51 paralogs. **(A**–**C)***Li*Rad51-3 and *Li*Rad51-6 bind preferentially tailed DNA over single-strand DNA. A mixture of ssDNA and tailed DNA (3′-tails, lanes 1–6 or 5′-tail, lanes 7–12) was incubated together with increasing concentration (0–100 nM) of *Li*Rad51-3 (A), *Li*Rad51-4 (B) and *Li*Rad51-6 (C). Error bars represent SD from three independent experiments.

**Figure 3.**
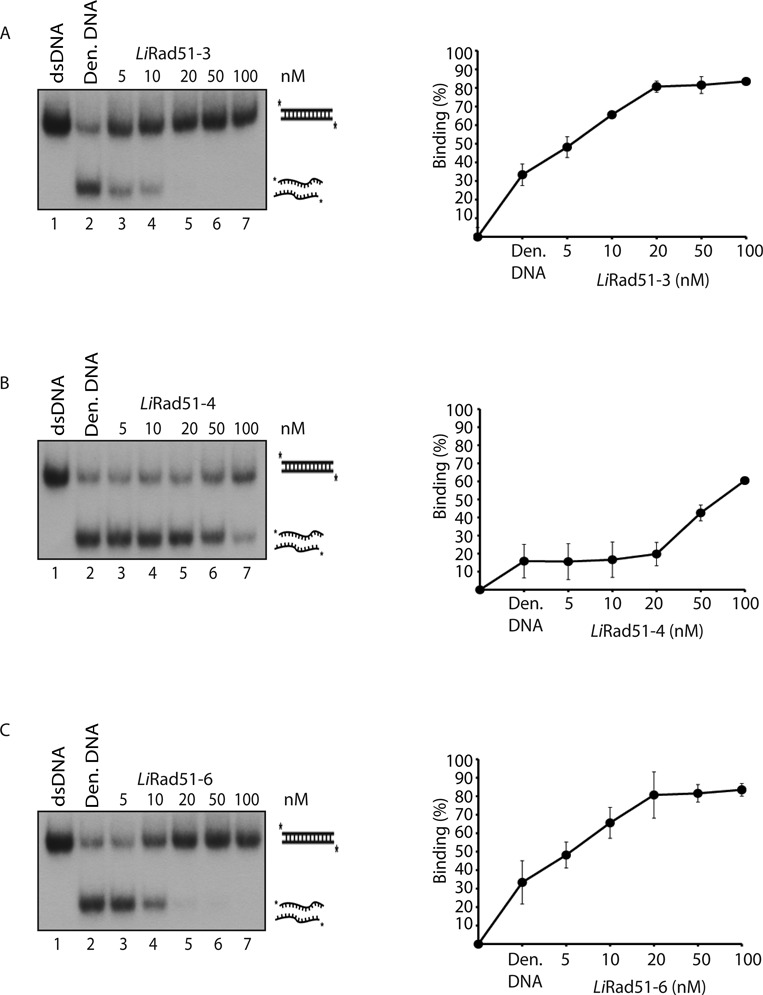
Annealing properties of purified *Li*Rad51 paralogs. **(A**–**C)***Li*Rad51-3 and *Li*Rad51-6 promote single-strand annealing. Lane 1, purified 63 pb duplex DNA. Reactions (lanes 2–7) contain denatured 63 pb duplex with an increasing concentration (0–100 nM) of *Li*Rad51-3 (A), *Li*Rad51-4 (B) or *Li*Rad51-6 (C). Error bars represent SD from three independent experiments.

**Figure 4.**
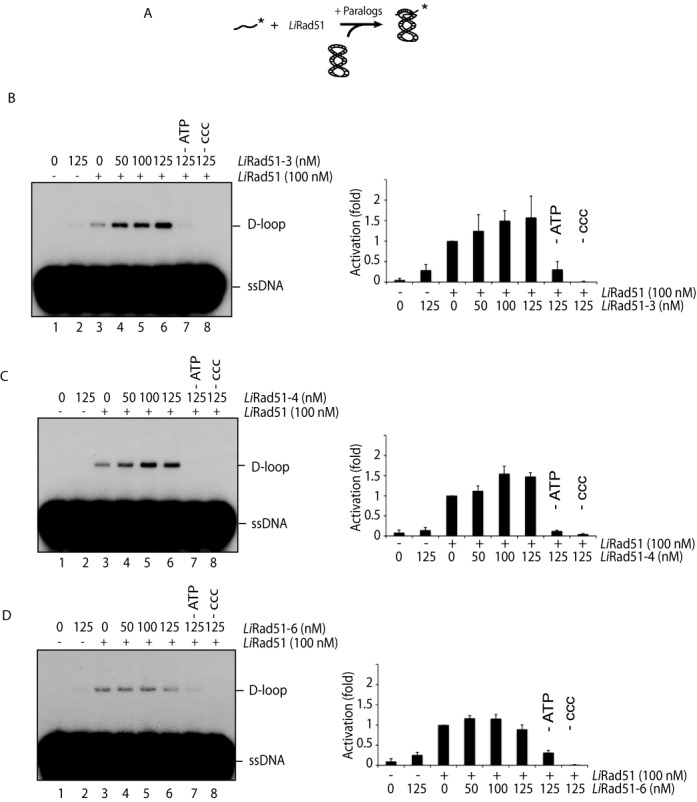
Stimulation of D-loop activity of *Li*Rad51 by the *Li*Rad51 paralogs. **(A)** Schematic representation of the D-loop assay using a labeled 100-mer single-strand DNA and supercoiled DNA template**. (B**–**D)***Li*Rad51-3 and *Li*Rad51-4 stimulate *Li*Rad51-catalysed D-loop formation. D-loop reactions contain DNA only (lane 1), *Li*Rad51 paralog alone (125 nM, lane 2), *Li*Rad51 (100 nM, lanes 3–6) with an increasing concentration (0–125 nM) of *Li*Rad51-3 (B), *Li*Rad51-4 (C) or *Li*Rad51-6 (D). Reactions 7 and 8 do not contain ATP or supercoiled DNA (ccc), respectively. Error bars represent SD from three independent experiments and data are measured in terms of fold activation compared to the control.

**Figure 5.**
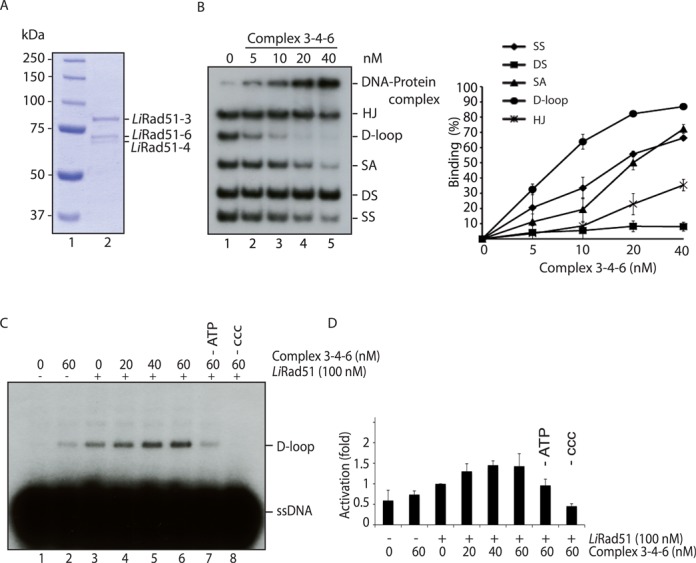
Purification, DNA binding and D-loop activity of purified 3-4-6 complex. **(A)** SDS-PAGE of purified 3-4-6 complex. Purified complex (300 ng) was loaded on a 8% SDS-PAGE and stained with coomassie blue. Lane 1, molecular weight markers (Bio-Rad); lane 2, purified 3-4-6 complex**. (B)** Purified 3-4-6 complex binds preferentially D-loop substrate. Competition EMSA was performed with increasing concentration of 3-4-6 complex (0–40 nM, lanes 1–5) with Holliday junctions (HJ), D-loop, splayed arm (SA), dsDNA and ssDNA on 8% acrylamide gel. The data shown in the graph are an average of three independent experiments. **(C)** 3-4-6 complex stimulates *Li*Rad51-catalysed D-loop formation. D-loop reactions contain DNA only (lane 1), 3-4-6 complex alone (60 nM, lane 2), *Li*Rad51 (100 nM, lanes 3–6) with an increasing concentration (0–60 nM) of 3-4-6 complex. Reactions 7 and 8 do not contain ATP or supercoiled DNA (ccc), respectively. **(D)** Error bars represent SD from three independent experiments and data are measured in terms of fold activation compared to the control.

**Figure 6.**
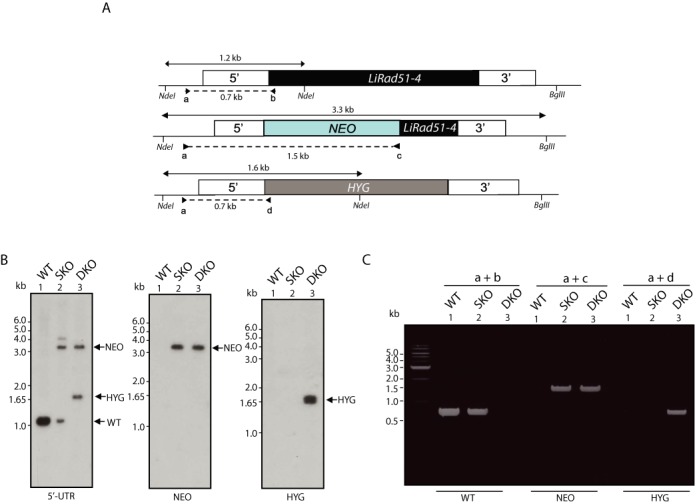
Inactivation of *LiRad51*-*4*.**(A)** Schematic representation of the *LiRad51*-*4* gene (top) and inactivation cassettes with the neomycin phosphotransferase (NEO, middle) and hygromycin phosphotransferase (HYG, bottom). The position of the primers pairs (a+b, a+c, a+d) used in (C) is depicted by arrows. **(B)** Southern blots of three different populations digested with *NdeI* et *BglII* after hybridization with 5′UTR (left), NEO (middle) and HYG (right) probes. DNA in lane 1 has no integration referred as *L.infantum* wild-type, in lane 2 has a NEO insertion in one allele and lane 3 has both NEO/HYG insertions, referred as double knock-out (DKO). **(C)** PCR analysis of three different populations after integration of the NEO and HYG cassettes on DNA from the cells described in (B).

### Southern blot analysis

Genomic DNA was isolated using DNAzol as recommended by the manufacturer's instructions (Invitrogen) and digested with the indicated restriction enzymes (NEB). DNA used for the probe result of PCR amplification from *Leishmania* genomic DNA or from plasmids containing markers hygromycin (HYG) or neomycin (NEO). 235 bp *MRPA* fragment was obtained by PCR from *Leishmania* genomic DNA. Southern blot hybridization were performed with [α-^32^P]dCTP-labeled DNA probes according to standard protocols (54).

### Growth curve and MMS treatment

Cultures containing 5 × 10^6^ cells of *Leishmania infantum* promastigotes were grown in SDM-79 medium supplemented with 10% heat-inactivated fetal bovine serum, 5 μg/ml hemin and 5 μM biopterin at pH 7.0 and incubated at 25°C for the indicated time. At each day, the OD_600nm_ was recorded. The same experimental conditions were used for Methyl methanesulfonate (Sigma-Aldrich) treatment and OD_600nm_ was taken when logarithm phase was reach (∼0,6).

### Purification of *Leishmania infantum* Rad51 paralogs and Rad51

*Li*Rad51 and *Li*Rad51 paralogs were purified from baculovirus-infected Sf9 cells as described in (55) and (51), respectively. For the expression of the complex 3-4-6, insect cells were infected for 72h with the *Li*Rad51-3- GST-His, *Li*Rad51-4-GST and *Li*Rad51-6-GST baculoviruses. Protein concentrations were determined by Bradford assays and by comparison with serially diluted calibrated BSA following Coomassie blue staining.

### Immunoprecipitation and pull-down assays

Immunoprecipitation from purified proteins were performed as follows. Purified *Li*Rad51 (500 ng) and *Li*Rad51-His paralogs (100 ng) were incubated in 100 μl of IPR buffer (20 mM Tris-Acetate pH 8.0, 125 mM KOAc, 0.5 mM EDTA, 0.5% NP40, 10% glycerol, 0.1 mg/ml BSA, 1 mM DTT and 2 mM ATP) for 30 min at 37°C. Two μg of anti-Histidine (Clontech) or control IgG monoclonal antibodies were added for 30 min at 4°C followed by the addition of 15 μl protein A/G sepharose beads (Pierce) for 30 min at 4°C. Beads were washed four times in IPR buffer containing 300 mM KOAc and proteins were eluted with 30 μl of Laemmli buffer. Immunoprecipitates were visualized by Western blotting using the polyclonal RAD51 pAb18 and monoclonal Histidine (Clontech) for *Li*Rad51 and *Li*Rad51-His paralogs, respectively.

Immunoprecipitation from Sf9 insect cells were performed as follows. Each *Li*Rad51-His paralog baculoviruses were individually co-infected with the baculoviruses *Li*Rad51-3-Myc, *Li*Rad51-4-V5 and *Li*Rad51-6-HA in order to immunoprecipitate the His-tagged protein. After two days at 27°C, the cell pellet was resuspended in 5 ml of IP100 buffer (20 mM Tris-Acetate pH 8.0, 100 mM KOAc pH 8.0, 0.5 mM EDTA, 0.5% NP40, 10% glycerol, 0.1 mg/ml BSA, 1 mM DTT) containing protease inhibitors PMSF (1 mM), aprotinin (0,019 UIT/ml) and leupeptin (1 μg/ml). The lysate was sonicated two times for 10 s with a Branson sonifier at 30% burst and centrifuged 10 min, 13 000 rpm at 4°C. Two μg of anti-Histidine (Clontech) or control IgG monoclonal antibodies were added to soluble extract (1 mg) and incubated for 2 h at 4°C. A volume of 15 μl of protein A/G sepharose beads (Pierce) was added followed by an 1 h period incubation at 4°C. Beads were washed four times with IP100 buffer containing 500 mM of KOAc and proteins were eluted with 30 μl of Laemmli buffer. Immunoprecipitates were visualized by Western blotting using the indicated antibodies.

The same protocol was performed for GST pull-down assays. Baculovirus *Li*Rad51-GST was co-infected for 48h with *Li*Rad51-3-Myc, *Li*Rad51-4-V5 and *Li*Rad51-6-HA baculoviruses and lysed in PBS buffer (300 mM NaCl, 1 mM EDTA, 1 mM DTT and 0,2% triton in PBS) containing protease inhibitors PMSF (1 mM), aprotinin (0,019 UIT/ml) and leupeptin (1 μg/ml). Soluble extracts were incubated during 1h at 4°C with 40 μl Glutathione S-transferase beads followed by four washes in PBS buffer containing 500 mM NaCl.

### D-loop assays

The D-loop assays with single-strand DNA JYM1413 (Supplementary Table S1) was conducted essentially as described (56). A ^32^P-labeled 100-mer oligonucleotide substrate (1 μM nucleotides) was incubated for 5 min with 100 nM of *Li*Rad51 at 37°C in 25 mM Tris-acetate pH 7.5, 1 mM DTT, 20 mM creatine phosphate, 5 U/ml phosphocreatine kinase, 2 mM ATP and 0.5 mM CaCl_2_ in 10 μl. The reaction was incubated 5 min with or without *Li*Rad51 paralogs at the indicated concentration. CsCl-purified pPB4.3 replicative form I DNA (300 μM) was added and the reaction was incubated for 60 min. One-fifth volume of stop buffer (20 mM Tris-Cl pH 7.5 and 2 mg/ml proteinase K) was added, followed by 20 min incubation at 37°C. Labeled DNA products were analyzed by electrophoresis through a 0.8% TAE1X /agarose gel run at 65V during 1h30, dried onto DE81 filter paper and visualized by autoradiography.

### DNA binding assays

DNA substrates were made by the annealing of the primer JYM945 for DS, JYM926 for SA, JYM926, JYM927, JYM928 for HJ with the ^32^P-labeled primer JYM925. D-loop structures were generated by the annealing of the primers JYM1395 and JYM1396 with the ^32^P-labeled primer JYM1745. The primer JYM699 for 3′-tail and JYM697 for 5′-tail were annealed with the ^32^P-labeled primer JYM696 (Supplementary Table S1). Reactions (10 μl) contained 25 nM of ^32^P-labeled DNA oligonucleotides and *Li*Rad51 paralog, at the indicated concentrations, in binding buffer (25 mM MOPS (morpholine-propanesulfonic acid) pH 7.0, 0,2% tween-20, 1 mM CaCl_2_ and 2 mM DTT). Proteins were added to reactions and incubated at 37°C for 10 min, followed by 15 min of fixation with 0.2% glutaraldehyde. Reactions were loaded onto an 8% TBE 1X acrylamide gel and run at 150V during 1 h 50 min.

### Single-strand annealing reactions

Reactions (10 μl) contained 63-mer denatured ^32^P-labeled double-strand DNA (70 nM) with *Li*Rad51 paralogs, at the indicated concentrations, in MOPS buffer (25 mM MOPS (morpholine-propanesulfonic acid) pH 7.0, 0,2% tween-20, 2 mM DTT and 1.5 mM Mg(CH_3_COO)_2_). To denature the DNA, the probe was boiled during 5 min then incubated 2 min on ice prior the addition of the proteins. Incubation was conducted at 20°C for 10 min followed by deproteinization by the addition of one-fifth volume of stop buffer (20 mM Tris-Cl pH 7.5 and 2 mg/ml proteinase K) for 10 min at 20°C. Labeled DNA reactions were analyzed by electrophoresis on an 8% TBE 1X PAGE gel, run at 150 Volts for 3 h, dried onto DE81 filter paper and visualized by autoradiography.

### Pulsed field gel electrophoresis

Intact chromosomes were prepared from late log phase culture cultures of *Leishmania* promastigotes and separated by pulsed-field gel electrophoresis using a Bio-Rad CHEF-DR III apparatus (Hercules, California, United States) as described (57).

### DNA preparation for sensitive PCR assays

Late log phase promastigotes (10 ml) were pelleted at 3000 rpm for 5 min, pellets were washed with Hepes-NaCl, resuspended in suspension buffer (100 mM EDTA, 100 mM NaCl, 10 mM Tris pH 8.0), and lysed in 1% SDS with 50 μg/ml proteinase K for 1 h at 37°C. The DNA was extracted with 1 volume of phenol, precipitated with 2 volumes of 99% ethanol, washed with 70% ethanol twice and dissolved in 500 μl TE. RNAse (20 μg/ml) was added and incubated for 30 min at 37°C, followed by addition of 50 μg/ml proteinase K and 0.1% SDS at 37°C for 30 min. DNA was extracted with 1 volume of phenol, precipitated and washed as above, then dissolved in Milli-Q water. DNA was quantified using a Nanodrop spectrophotometer.

### Semi-quantitative polymerase chain reaction

All putative recombination events on the genome sequence of *L. infantum* JPCM5 were identified by bioinformatics analyses as described (48). The PCR products, which needed to be longer than the size of the repeats used for recombination, required optimizations. PCR reaction mixture for amplicons consisted of 100 ng of phenol-purified genomic DNA isolated as described above, 0.4 μM of forward (P2a or P3a) and reverse (P2b or P3b) primers (Supplementary Table S1), 0.2 mM dNTPs, 1,5 U of Phusion (New England Biolabs) for amplicon MRPA-R2 or 1.25 U of FastStart Taq DNA polymerase (Roche) for amplicon MRPA-R3, 1 × GC buffer (MRPA-R2) or 1 × PCR buffer + MgCl_2_ (MRPA-R3), 0.75 μl DMSO (MRPA-R2) or 3.3 mg/ml BSA (MRPA-R3). The total reaction mixture was made up to 25 μl by addition of the genomic DNA. For each PCR reaction, the annealing temperature was optimized as well as the number of cycles to prevent saturation of the amplification. The housekeeping gene *GAPDH* was used as a control to normalize the amount of DNA loaded in each reaction. Saturation of band intensities of the amplified PCR products was determined using the AlphaImager 2000 software. Densitometric analyses were performed using ImageJ and Agfa Arcus 2 scanner.

## RESULTS

### Purification of *Leishmania infantum* Rad51 paralogs

To study the biochemical role of *Li*Rad51 paralogs, the *Li*Rad51-3, *Li*Rad51-4, *Li*Rad51-6 tagged fusion proteins were separately expressed using the baculovirus system where each protein was purified from Sf9 insect cells by two-step affinity purification. The long PreScission cleavable GST tag (29 kDa) flanks the protein of interest in N-terminus while a 10XHis-tag is at the C-terminus of the protein, which enables a second affinity purification step. Soluble extract expressing GST-*Li*Rad51 paralog-His were incubated with GST beads followed by GST cleavage using PreScission protease. The eluted protein material was then added on Talon beads for affinity purification using the C-terminal His-tag. We succeed to purify each protein to >95% homogeneity. We quantified the purified proteins to use the same concentration of protein in the experiments described below (Figure 1A).

### Interaction between *Li*Rad51 paralogs

Immunoprecipitation analyses were then carried out using two approaches to validate the interaction between *Li*Rad51 and individual *Li*Rad51 paralog proteins. First, purified proteins were used to assess direct interaction without the requirement of other factors and, secondly, pull-down assays with overexpressed protein in Sf9 insect cells. When the His-tagged purified *Li*Rad51 paralogs were incubated with purified *Li*Rad51, we observed a co-complex between *Li*Rad51 and each paralog (Figure 1B). To perform reciprocal pull-down, we next engineered constructs for each paralogs with different tags (*Li*Rad51-3-Myc, *Li*Rad51-4-V5 and *Li*Rad51-6-HA) and co-expressed these fusion proteins individually with *Li*Rad51-GST in insect cells. As expected, we detected each paralog following a GST pull-down while the pull-down control overexpressing only the paralog revealed no band at the expected molecular mass, consistent with an absence of binding to the GST beads (Supplementary Figure S1A).

The interactions between the paralogs were studied further, taking advantage of the His-tagged baculovirus and the *Li*Rad51-3-Myc, *Li*Rad51-4-V5 and *Li*Rad51-6-HA constructs. Following an anti-His IP, we observed that all proteins interacted together and with themselves except for of *Li*Rad51-3 (Supplementary Figure S1B).

### *Li*Rad51 paralogs bind preferentially tailed DNA and D-loop structure

Based on previous works on RAD51-like members, we hypothesized that *Leishmania* RAD51 paralogs display DNA-binding ability. Therefore, by using electrophorectic mobility-shift assay, we examined the substrate preference of these proteins for ssDNA in competition with tailed duplex DNA. Our observations indicate that *Li*Rad51 paralogs preferentially bind ssDNA tail overhangs over ssDNA (Figure 2). At 100 nM, *Li*Rad51-3 and *Li*Rad51-6 show higher affinity for tailed DNA (70% of DNA binding) compared to *Li*Rad51-4 (40% of DNA binding) (Figure 2). To further evaluate DNA-binding properties of *Li*Rad51 paralogs, we tested in competition other structures, which are found during the HR process (Holliday jonction (HJ), D-loop) and replication (splayed arm (SA)). The binding profile obtained suggests a strong affinity for D-loop structure, consistent with the predicted role of paralogs in strand invasion (Supplementary Figure S2). The use of multiple DNA constructs in the same competitive EMSA experiment followed by glutaraldehyde fixation resulted in a sharp shifted band. To confirm if this band represented binding of the protein with DNA, we performed DNA binding assays with each substrate and the reaction was loaded on an agarose gel (Supplementary Figure S3). However, no stimulatory effect was found on *Li*Rad51 DNA binding with the paralogs (data not shown). Taken together, these results indicate that individual *Li*Rad51-3, *Li*Rad51-4 and *Li*Rad51-6 exhibit DNA binding preferentially for tailed DNA duplex and D-loop structure.

### Single-strand annealing and D-loop formation activities of *Li*Rad51 paralogs

As part of further biochemical characterization of *Li*Rad51 paralogs, we tested the capacity of these proteins to anneal complementary single-strand DNAs. For this purpose, we denatured a 63-mer dsDNA for generating two ssDNAs, which were incubated with the proteins. To detect annealing activity, deproteinized reactions were migrated on a polyacrylamide gel and we examined the formation of dsDNA products. We noticed that *Li*Rad51-3 and *Li*Rad51-6 catalyzed annealing while *Li*Rad51-4 was less efficient (Figure 3). Even at five-fold the concentration (100 nM), ssDNA remained in *Li*Rad51-4 reactions (Figure 3B, lane 7) while in *Li*Rad51-3 or *Li*Rad51-6 (20 nM) reactions, renatured DNA products were completely formed (Figure 3A–C, lane 5).

Following the formation of the nucleofilament on DNA, RAD51 is known to promote strand invasion and form D-loop structure. To test this, we incubated a 5′-labeled ssDNA coated with *Li*Rad51 with a supercoiled plasmid containing a homologous sequence (Figure 4A). Thus, we investigated if *Li*Rad51 paralogs can stimulate strand invasion triggered by *Li*Rad51 (55). Remarkably, in condition where *Li*Rad51 activity is minimal (100 nM), purified *Li*Rad51-3 or *Li*Rad51-4 were able to stimulate D-loop production in a concentration dependent manner, while *Li*Rad51-6 could not (Figure 4B–D). Reaching at least 1.5-fold (Figure 4B and C, lane 6), this activation is *Li*Rad51 and ATP dependent (Figure 4B and C, lanes 2 and 7). Using three buffers conditions, we did not observe strand activity for the *Li*Rad51 paralog proteins on their own (Supplementary Figure S4). Since the *Li*Rad51-3 and *Li*Rad51-6 perform DNA annealing and *Li*Rad51-3 and *Li*Rad51-4 can stimulate strand invasion, these results suggest that *Li*Rad51 paralogs are distinct from each other where *Li*Rad51-3 might be at the heart of this key step in HR.

### Purification, DNA binding and stimulation of strand invasion by 3-4-6 complex

In line with the existence of complexes between human paralogs (15), we aimed to purify the *Li*Rad51 paralogs in complex to confirm the nature of this association. Using a baculovirus expression system, we co-expressed all three GST-tagged paralogs in Sf9 insect cells where *Li*Rad51-3 also carried a histidine tag. We proceed by two-step affinity purification (GST beads followed by Talon beads) and succeed to copurify to homogeneity the complex of *Li*Rad51-3, *Li*Rad51-4, *Li*Rad51-6 (termed 3-4-6 (Figure 5A)). We have performed two additional experiments to show that the three paralogs interact as a complex. First, we performed a GST pull-down with co-expressed LiRad51-3-GST, LiRad51-4-V5 and LiRad51-6-HA baculoviruses and cleaved the GST, removing the possibility that the complex is facilitated by GST dimerization. Second, we performed immunoprecipitation using anti-HA antibody on Sf9 cells extract containing *Li*Rad51-3-Myc, *Li*Rad51-6-HA and *Li*Rad51-4-V5. In both cases, we detected the three Rad51 paralogs together (Supplementary Figure S5).

We next sought to determine whether the 3-4-6 complex was active like the individual proteins. We performed DNA binding assays with different substrates in competition (ssDNA, dsDNA, D-loop, SA, HJ). As expected, the complex harbors affinity for D-loop, SA and ssDNA (Figure 5B) and is also capable of stimulating *Li*Rad51 D-loop formation like the individual components (Figure 4 and 5C-D).

### Inactivation of *Li*Rad51-3 and *Li*Rad51-4 genes

It has been previously observed that formation of extrachromosomal circular amplicons is facilitated by the *Li*Rad51 recombinase (48). However, in this study, two out of seven *Li*Rad51 mutants selected for arsenite resistance still form circular amplicons suggesting alternative mechanisms. Because of their ascribed roles in homologous recombination, we hypothesized that the *Li*Rad51 paralogs could be the factors promoting extrachromosomal circular amplicons. To test this, we attempted to inactivate *LiRad51*-*3* (closest to RAD51C, (50)), *LiRad51*-*4* (XRCC2, (49)) or *LiRad51*-*6* (RAD51D, (49)) through gene disruption by double crossed-over. Inactivation of both alleles by the integration of neomycin phosphotransferase (NEO) and hygromycin phosphotransferase (HYG) markers was possible for the *LiRad51*-*4* gene. After two rounds of electroporation, Southern blot has been performed on genomic DNA digested by BglII and NdeI enzymes. Using a complementary probe to 5′UTR region, WT cells yielded as expected a 1.2 kb band while a 3.3 kb and 1.6 kb bands for NEO and HYG respectively are observed in null mutant (Figure 6A and B). These results were also confirmed by PCR where no more WT copy was detected in double-knockout cells (Figure 6C, Supplementary Figure S6). Unexpectedly, we did not succeed to generate *LiRad51*-*3* or *LiRad51*-*6* null mutants, using this strategy.

As an alternative strategy to generate *LiRad51*-*3* or *LiRad51*-*6* null mutants, we transfected an episomal vector (pSP72α*PURO*α) harboring the gene targeted in single knockout cells to allow expression of the protein during disruption of the second allele. Loss of both copies of *LiRad51*-*3* gene was achieved with this strategy (Supplementary Figure S7). Digestion with *NdeI* and *NcoI* yielded a 1.8kb (for WT copy), 0.6 kb (for HYG) and 0,8 kb (for NEO) fragments when hybridized to 5′-UTR probe (Supplementary Figure S7A and C). PCR analysis with specific primers validates also this result (Supplementary Figure S7A and B). With the aim to get rid of the *Li*Rad51-3 episomal construct in the double knockout parasites, we removed puromycin selection for 30 passages. Surprisingly, instead of having a reduction in the copy number of the vector over time, we clearly see an increase as Southern blot and PCR results showed for passages 15 and 30 (Supplementary Figure S7D and E), a phenomenon usually associated with genes reputed to be essential in *Leishmania*. Many attempts using different strategies were tried to inactivate *LiRad51*-*6* gene but without success (data not shown).

### Phenotypes of *LiRad51*-*4* null mutant

**Figure 7.**
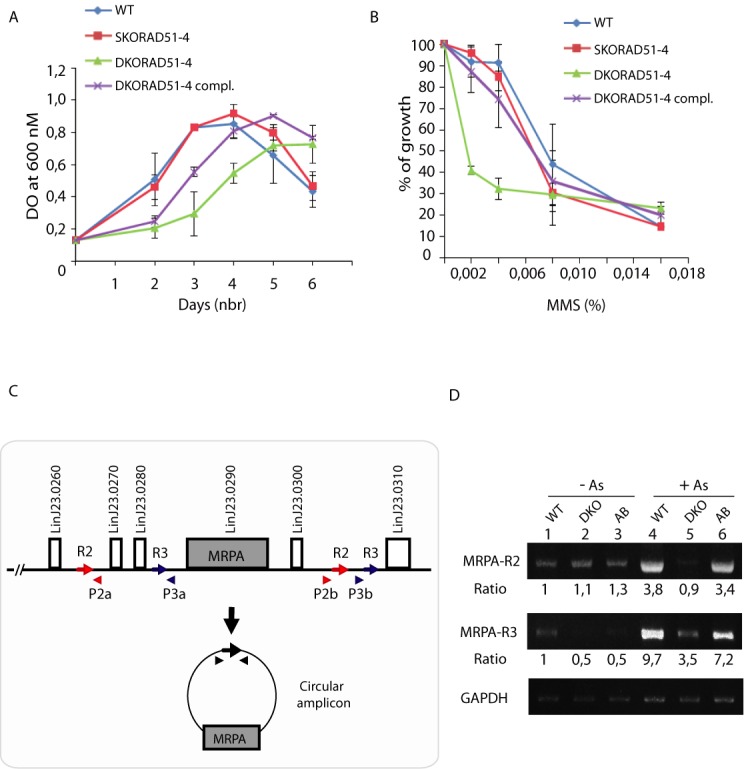
Phenotypic analyses of DKO*LiRad51*-*4*.**(A)** Growth curve of DKO*LiRad51*-*4* grown as promastigote in SDM over time. *L.infantum* wild-type (circles), SKO*LiRad51*-*4* (squares), DKO*LiRad51*-*4* (triangles) and DKO*LiRad51*-*4* complemented with a plasmid expressing DKOLiRad51-4 (cross) are showed. Error bars represent SD from three independent experiments. **(B)** DKO*LiRad51*-*4* displays MMS sensitivity. Growth retardation in presence of MMS was measured for the cultures described in (A). Error bars represent SD from three independent experiments. **(C)** Schematic representation of the *MRPA* locus and the circular amplicon generated by HR between specific repeats (repeat 2 (R2) represented by red arrows or repeat 3 (R3) by blue arrows) on chromosome 23 in *L. infantum*. **(D)** Adaptive gene amplification in DKO*LiRad51*-*4* parasites upon drug selection. *L. infantum* WT (1, 4), DKO*LiRad51*-*4* (2, 5) and DKO*LiRad51*-*4* compl. (AB) (3, 6) were either cultured in the absence of drug (-) or in the presence of 80 μM arsenite for three passages. Total DNA was extracted from cells and semi-quantitative PCRs were performed to detect *MRPA-*R*2* or *MRPA-*R*3* circular amplicons. P2a/P2b and P3a/P3b are respectively the primers used for detecting the rearrangements. Densitometric ratios of PCR band intensities are indicated at the bottom. One representative experiment out of three is shown. Amplification of the chromosomal *GAPDH* gene was used as loading control.

**Figure 8.**
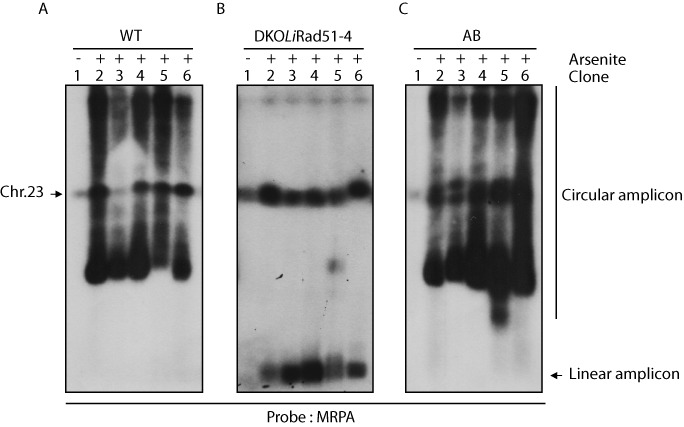
***M****RPA* locus amplifications upon arsenite selection and the role of the DKO*LiRad51*-*4*. Rearrangements that occurred in *L. infantum* WT cells (A), in DKO*LiRad51*-*4* (B) and in DKO*LiRad51*-*4* compl. (AB) (C) all selected for arsenite resistance (up to 160 μM = 4x EC_50_). The DNA of resistant clones were separated by pulse field gel electrophoresis and hybridized to a *MRPA* probe. Lane 1, unselected population, lanes 2–7 independent arsenite resistant clones. Chromosome 23, linear and circular amplicon are represented.

We tested if *LiRad51*-*4* null mutant triggers growth defect and sensitivity to alkylating damaging agent methyl methanesulphonate (MMS). Consistent with the fact that *LiRad51*-*4* is involved in DNA damage repair, we observed a growth delay and MMS sensitivity for double knockout cells (Figure 7A and B). Introduction of an episomal vector coding for *Li*Rad51-4 protein restored partially the growth compared to the WT and reversed totally the MMS sensitivity phenotype (Figure 7A and B). We next hypothesized that *Li*Rad51-4 may have a role in formation of extrachromosomal circular amplicons upon drug selection. To test this, we used a sensitive PCR assay where specific primers are designed near two homologous direct repeat sequences to allow detection of novel junctions produced after genomic rearrangement (41). By selecting wild-type, DKO*Li*Rad51-4 and reverted mutant (complemented with the *Li*Rad51-4 wild-type gene) for arsenite (As) resistance, we monitored rearrangements and amplification of the ABC protein MRPA (58). As depicted in Figure 7C, we selected two different set of direct repeats (2 and 3), which border the *MRPA* gene to analyze circular gene amplification. We detected an increase of PCR products upon drug selection (2x EC_50_) for wild-type (Figure 7D, lanes 1 and 4) and complemented cells (Figure 7D, lanes 3 and 6) but not for DKO*Li*Rad51-4 (Figure 7D, lanes 2 and 5) in both cases. High arsenite resistant clones were also obtained *in vitro* after five passages (4X EC_50_). DNA were separated by pulse-field gels then hybridized with *MRPA* probe. Hybridization patterns of five resistant clones of DKO*Li*Rad51-4 show no clone with *MRPA* circles (Figure 8B) while wild-type and revertant cells exhibit circular amplification in all clones (Figure 8A and C). In contrast, we can also observe that all DKO*Li*Rad51-4 mutants generate linear amplicons (Figure 8B). Our findings give striking evidence for the role of *Li*Rad51-4 in the formation of extrachromosomal circles.

## DISCUSSION

Parasitic diseases have an enormous health impact and the burden of these diseases is exacerbated by parasite drug resistance. The unresponsiveness to treatments affect a large portion of infected individuals, suggesting several resistance pathways. We have shown recently that *Leishmania infantum* Rad51 is involved in genome rearrangement by producing circular amplicons through recombination of direct repeated sequences (48). This key player of homologous recombination is however not essential for this process since a few DKO*Li*Rad51 resistant mutants still generated MRPA circles to resist arsenite selection. In order to better understand this adaptive mechanism, the goal of this study was to find whether other recombination proteins could trigger circular amplicons to promote drug resistance.

From extensive studies on yeast and mammalian homologous recombination pathways, a model emerged where key genes have been identified and biochemically characterized (8). Among them, the recombinase RAD51 and its cofactors, BRCA2, PALB2, RAD52 and RAD51 paralogs enable the core reaction consisting of the homology search and DNA strand invasion. With the aid of bioinformatics and genomic sequences, it is possible to target homologs of recombination enzymes in Trypanosomatidae. Identified in *T.brucei, T.cruzi, L.major* and *L.infantum*, Rad51 shares between 60% and 80% sequence identity with its human counterpart while Brca2 from parasites possesses critical features as an OB fold for DNA binding and BRC repeats for Rad51 interaction (49). The PALB2 and RAD52 mediators have not been detected by homology in Trypanosomatidae (49). We reported that *Li*Brca2 acts as mediator to facilitate the displacement of RPA and stimulate *Li*Rad51 D-Loop formation (55). It is perhaps surprising that no effect on the generation of circular amplicons has been observed in a *Li*Brca2 mutant (unpublished data), since *Li*Rad51 has been found in the cytoplasm. This suggests that other nuclear recombination proteins can compensate the loss of nuclear function of *Li*Rad51. In particular, three possible candidates are three *L.infantum Rad51 paralogs* with conserved residues for Walker A and B motifs. Rad51-3 is closest to RAD51C, Rad51-4 to XRCC2 and Rad51-6 to RAD51D (49). Thus, the biochemical and genetic characterization of these latter proteins has been undertaken.

This report shows that individual purification of full-length *Leishmania* Rad51 paralog proteins is achievable through a two-step affinity purification. Consistent with the fact that human RAD51 paralogs interact with the recombinase RAD51, we used pull-down experiments to show direct interaction between each paralog and *Li*Rad51. These data suggest that the single paralogs could individually act on the formation of the *Li*Rad51 presynaptic filament. Hence, the protein–protein interaction observed is also in line with the requirement of RAD51 paralogs for RAD51 foci formation in mammalian cells (10). In a competition assay, all three proteins unveiled strong affinity for D-loop substrate and single-strand DNA. Following double-strand break, DNA is nucleolytically processed to form a single-strand DNA with an overhang tail to serve ultimately as a structure to invade intact sister chromatid and form joint molecules (D-loops). Our data show that paralogs have a preference for tailed duplex DNA over single-strand DNA implying a role during strand invasion.

Interestingly, there is some overlap between our findings with *T. brucei* Rad51 paralogs (50). *T. brucei* Rad51-3 also appears to be the central repair paralog, perhaps explaining why it is essential in *Leishmania*. *T. brucei* Rad51-4 appears to play a lesser role in repair than the other paralogs, consistent with lowered activities of this *Leishmania* factor relative to the others. Furthermore, *T. brucei* Rad51-5, which is absent in *Leishmania*, does not appear to interact with 3-4-6 suggesting that it might has evolved to play another role.

According to *Leishmania* DNA amplification model, recombination among direct repeated sequences requires (1) the search for the homology sequence and (2) the annealing between repeats (41). Thus far, *Li*Rad51 is the only protein in Trypanosomatidae reported to catalyze homology search and strand invasion in presence of ATP (55). This finding prompted us to ask whether Rad51 paralogs can mediate D-loop formation or/and enhance *Li*Rad51 D-loop formation activity. By using an *in vitro* recombination assay, we observed that *Li*Rad51-3 and *Li*Rad51-4 can stimulate *Li*Rad51 activity while none of the paralogs could invade DNA on its own, under our experimental conditions. This suggests that *Li*Rad51 is the main protein, able to scan DNA and find homology. Here, we cannot exclude the possibility that a yet-to-be discovered recombinase (e.g. *Li*Dmc1) is entailed in the formation of circular amplicon and has sustained resistance in the DKO*Li*Rad51 mutants. Therefore, we tested if the paralogs could trigger annealing of single-strand DNA. We observed that *Li*Rad51-3 and *Li*Rad51-6 harbor this activity. Compared to the others paralogs, *Li*Rad51-4 has weaker DNA affinity for ssDNA (Figure 2) and displays a weak annealing activity (Figure 3). These data imply that each paralog has an impact on *Li*Rad51 activity either on homology search (*Li*Rad51-3/4) and/or strand annealing (*Li*Rad51-3/6). Divergent features among paralogs might explain why *Leishmania* conserved three paralogs through evolution to accomplish different tasks. In comparison to *Trypanosoma Brucei* where four RAD51 paralogs have been identified, all these latter proteins act in recombination and RAD51 dynamic but not all equivalently (50). Based on findings pertaining two complexes present in mammalian cells (BCDX2 and CX3), it is tempting to speculate that these paralogs could form a complex and act in concert since they interact with each other. Thus, we purified the complex 3-4-6 and assessed DNA binding and strand invasion assays. Indeed, they do not hamper each other but function together.

Previous work on drug resistance in *Leishmania* highlighted key genes (e.g *DHFR-TS, MRPA*) bordered by repeats and overexpressed through DNA amplification during selective drug pressure (41). To better understand this protective mechanism, we attempt to generate *LiRad51 paralogs* null mutants by gene disruption in order to monitor circular DNA amplification. In all three proteins, removal of one single allele has been achieved but the second round of inactivation was only possible for *LiRad51*-*4*. Actually, this gene disruption failure has been often reported for essential genes in *Leishmania*. For example, attempts to inactivate the dihydrofolate reductase-thymidylate synthetase (*DHFR-TS*) or the gamma-glutamylcysteine synthetase (*GSH1*) genes led to the generation of extra copy maintaining the WT allele (57,59). After several efforts to abolish *LiRad51*-*3* and *LiRad51*-*6*, we transfected an episomal vector of the targeted gene in the single knockout to preserve temporally a gene copy through puromycin selection. Using this approach, we obtained a *LiRad51*-*3* mutant whereby the second selectable marker has been integrated at the homologous locus. Upon chromosomal gene inactivation we removed drug selection and even after 30 passages, the parasite still retained the episomal copy. It is salient to point out that *Leishmania* loose amplified DNA when the selection is removed suggesting that *LiRad51*-*3* might be essential (48). Using a similar approach, the *LiRad51*-*6* mutant could not be obtained and extensive additional work would be required to determine whether this gene is essential or not. Remarkably, we noticed that *LiRad51*-*4* mutants selected for arsenite resistance do not yield circular amplicons, suggesting that *LiRad51*-*4*, like *LiRad51*, is involved in the formation of extrachromosomal circles. To some extent, *Li*Rad51 paralogs overlap with *Li*Rad51 to bind DNA and trigger recombination. Altogether, our results provide mechanistic insights into the functions of parasite proteins normally known to mend DNA damage. Further studies are required to fully understand how this knowledge may be exploited to increase efficiency of current treatments and prevent resistance, although, some levels of resistance still emerges in the absence of *LiRad51* or *LiRad51*-*4*. A possible strategy to avoid resistance would be to inactivate *LiRad51* and/or *LiRad51*-*4*. While *Li*Rad51-4 inhibitors are yet to be discovered, it would be worthwhile to use RAD51 inhibitors (60,61) and see whether they can also be functional in the parasites and affect the emergence of drug resistance.

## SUPPLEMENTARY DATA

Supplementary Data are available at NAR Online.

SUPPLEMENTARY DATA
