# Supplementary material for: Roles of Rad51 paralogs for promoting homologous recombination in Leishmania infantum
Source: Nucleic Acids Res. 2015 Feb 24;43(5):2701–15. doi: 10.1093/nar/gkv118 (PMC4357719; doi:10.1093/nar/gkv118)
Supplement: SUPPLEMENTARY DATA [file supp_43_5_2701__index.html]

Roles of Rad51 paralogs for promoting homologous recombination in Leishmania infantum — SUPPLEMENTARY DATA 

# Roles of Rad51 paralogs for promoting homologous recombination in *Leishmania infantum*

## SUPPLEMENTARY DATA

**Files in this Data Supplement:**

- SUPPLEMENTARY DATA
