# Supplementary material for: Roles of Rad51 paralogs for promoting homologous recombination in Leishmania infantum
Source: Nucleic Acids Res. 2015 Feb 24;43(5):2701–15. doi: 10.1093/nar/gkv118 (PMC4357719; doi:10.1093/nar/gkv118)
Supplement: SUPPLEMENTARY DATA [file supp_gkv118_nar-00060-d-2015-File010.pdf]

Supplementary data

Roles of Rad51 paralogs for promoting homologous recombination in *Leishmania infantum*

Marie-Michelle Genois[1,2,3], Marie Plourde[3], Chantal Éthier[2,4], Gaétan Roy[3], Guy G. Poirier [2,4], Marc Ouellette[3] and Jean-Yves Masson[1,2,5]

[1] Genome Stability Laboratory, CHU de Québec Research Center, HDQ Pavilion, Oncology Axis, 9 McMahon, Québec City, QC G1R 2J6, Canada

[2] Department of Molecular Biology, Medical Biochemistry and Pathology, Laval University, Québec City, QC G1V 0A6, Canada

[3] Centre de Recherche en Infectiologie, CHUL, 2705 boul. Laurier, Quebec, Quebec G1V 4G2, Canada

[4] CHU de Québec Research Center, CHUL Pavilion, Oncology Axis, 2705 boul. Laurier, Quebec city, Quebec, G1V 4G2, Canada

Supplementary Table 1. Primers used in this study

Primer name	Sequence (5'-3')
JYM696	GGGCGAATTGGGCCCGACGTCGCATGCTCCTCTAGACTCGAGGAATTCGGTACCCCGGGTTCGAAATCGATAAGCTTACAGTCTCCATTTAAAGGACAAG
JYM697	CTTGTCCTTTAAATGGAGACTGTAAGCTTATCGATTTCGAACCCGGGGTA
JYM699	CCGAATTCCTCGAGTCTAGAGGAGCATGCGACGTCGGGCCCAATTCGCCC
JYM925	GGGTGAACCTGCAGGTGGGCAAAGATGTCCTAGCAATGTAATCGTCAAGCTTTATGCCGT
JYM926	ACGCTGCCGAATTCTACCAGTGCCAGCGACGGACATCTTTGCCCACCTGCAGGTTCACCC
JYM927	ACGGCATAAAGCTTGACGATTACATTGCTACATGGAGCTGTCTAGAGGATCCGACTATCG
JYM928	CGATAGTCGGATCCTCTAGACAGCTCCATGGTCGCTGGCACTGGTAGAATTCGGCAGCGT
JYM945	ACGGCATAAAGCTTGACGATTACATTGCTAGGACATCTTTGCCCACCTGCAGGTTCACCC
JYM1395	GCCAGGGACGGGGTGAACCTGCAGGTGGGCGGCTGCTCATCGTAGGTTAGTATCGACCTATTGGTAGAATTCGGCAGCGTCATGCGACGGC
JYM1396	GCCGTCGCATGACGCTGCCGAATTCTACCACGCTACTAGGGTGCCTTGCTAGGACATCTTTGCCCACCTGCAGGTTCACCCCGTCCCTGGC
JYM1413	GGGCGAATTGGGCCCGACGTCGCATGCTCCTCTAGACTCGAGGAATTCGGTACCCCGGGTTCGAAATCGATAAGCTTACAGTCTCCATTTAAAGGACAAG
JYM1745	AAGATGTCCTAGCAAGGCACCCTAGTAGC
JYM1791	ATGAAAAAGCCTGAACTCACC
JYM1797	ATGATTGAACAAGATGGATTG
JYM2119	cgagcacagacaaaagccaac
JYM2120	agacgtcgcggtgagttcaggctttttcatgcgggagttgacgaagagag
JYM2121	tgctcgaatacgtggaacgcgtcggcttttctattcctttgccctcgga
JYM2122	aaaagccgacgcgttccacgt
JYM2123	gacagttttgtaggcagtgtg
JYM2124	acccgcatgcgctgttccccttt
JYM2126	aggtcggcagctgttgctgctgtgatgaca tcagaagaactcgtcaagaag
JYM2127	tgtcatcacagcagcaacagc
JYM2128	aagaacgagtggaggagcgag
JYM2129	aataggttgatcgcatcacag
JYM2130	agacgtcgcggtgagttcaggctttttcat cgacatggatgcgccttggggg
JYM2131	ggtcatcacacatgcacgcgcacttccccg ctattcctttgccctcgga

JYM2132	cggggaagtgcgcgtgcatgtgt
JYM2133	gaaagcagtcggactggaagg
JYM2134	gtcactgcccagcagcggtgc
JYM2136	gggtcatctgagcacgacaaggacgtcggaa tcagaagaactcgtcaagaag
JYM2137	ttccgacgtccttgtcgtgct
JYM2138	aggtagcggcaagtgagatt
JYM2212	CCAGGGGCCCCTGGGGTACCAGATGGCACTGATCGAGTGCGCCG
JYM2213	CGTGGCACCAGAGCGAGCTCCTTATCGTCGTCATCCTTGTAATCTTC GCGCGATTCTCTGGCGCC
JYM2216	CCAGGGGCCCCTGGGGTACCAGATGATGATGTCCAGCACAGAT
JYM2217	CGTGGCACCAGAGCGAGCTCCTTATCGTCGTCATCCTTGTAATCTAG ATACAAGAAGGATGGCA
JYM2218	CCAGGGGCCCCTGGGGTACCAGATGAGCGCCACAGTGCCTGCT
JYM2219	CGTGGCACCAGAGCGAGCTCCTTATCGTCGTCATCCTTGTAATCTGC GTCTTCGATGCCGTGCTC
JYM2226	AAAGGCCTACGTCGACATGAGCGCCACAGTGCCTGCT
JYM2227	ACTTCTCGACAAGCTTTCAAGCGTAATCTGGAACATCGTATGGGTATG CGTCTTCGATGCCGTGCTC
JYM2228	AAAGGCCTACGTCGACATGGCACTGATCGAGTGCGCC
JYM2229	ACTTCTCGACAAGCTTCTACAGATCCTCTTCAGAGATGAGTTTCTGCT CTTCGCGCGARRCTCTGGCGCC
JYM2230	AAAGGCCTACGTCGACATGATGATGTCCAGCACAGAT
JYM2231	ACTTCTCGACAAGCTTTTACGTAGAATCGAGACCGAGGAGAGGGTTA GGGATAGGCTTACCTAGATACAAGAAGGATGGCAC
JYM2391	acctgcgtgcaatccatcttgttcaatcatgcgggagttgacgaagagag
JYM2392	acctgcgtgcaatccatcttgttcaatcatcgacatggatgcgccttggggg
JYM2774	CGGGGGATCCTCTAGAATGATGATGTCCAGCACAGAT
JYM2775	CGAGCAGCTGAAGCTTTTATAGATACAAGAAGGATGG
JYM2832	GTGCATCTGTGCTGGACATCATCAT
JYM2834	AGGCGGACGTGGATCTGGCAAAGG
JYM3083	agacggtgctgtgtgccttggtgga
JYM3084	ACTCGGCGCACTCGATCAGTGCCAT
JYM3085	tcgcggtgagttcaggctttttcat
MOUC2	ATGACCGAGTACAAGCCCAC
MOUD2	ACAGAACTGTCTCTGCTGCCTCAGGCACCGGGCTTGCGGG
P2a	GTGCGAAGGACCACACGACG
P2b	GTGGCACTGTCCTGTCATCG
P3a	TCCACGTGAGCATTCTGCCACA
P3b	CGGCAGCAAGAGTCCACATCCG

Supplementary Materials and Methods

Complex pull-down

The complex was expressed in Sf9 cells with *Li*Rad51-3-GST-His, *Li*Rad51-4-V5 and *Li*Rad51-6-HA baculoviruses for 72h. GST pull-down was performed as described in (51) and the proteins were visualized by Western blotting using the indicated antibodies (anti-Histidine (Clontech), anti-V5 (Fisher), anti- HA 16B12 (Covance)).

Immunoprecipitation using anti-HA antibody was performed on Sf9 cells extracts infected with *Li*Rad51-3-Myc, *Li*Rad51-6-HA and *Li*Rad51-4-V5 baculoviruses for 72h. The cell pellet was resuspended in 20 ml of IP150 buffer (50 mM Tris-HCl pH 7.5, 500 mM NaCl, 0.5% NP40) containing protease inhibitors PMSF (1 mM), aprotinin (0,019 UIT/mL) and leupeptin (1 µg/mL). The lysate was sonicated 3 times for 30 seconds with a Branson sonifier at 30 % burst and centrifuged 20 min, 13 000 rpm at 4°C. 40 µg of anti-HA 16B12 (Covance) were added to soluble extract (140 mg) and incubated for 2 hours at 4°C. A volume of 100 µL of protein A/G sepharose beads (Pierce) was added followed by an 1 hour period incubation at 4°C. Beads were washed two times with IP150 buffer and proteins were eluted with 50 µg of peptide HA in PBS overnight. Proteins were visualized by Western blotting using the indicated antibodies (anti-Myc Cell Signaling), anti-V5 (Fisher), anti- HA 16B12 (Covance)).

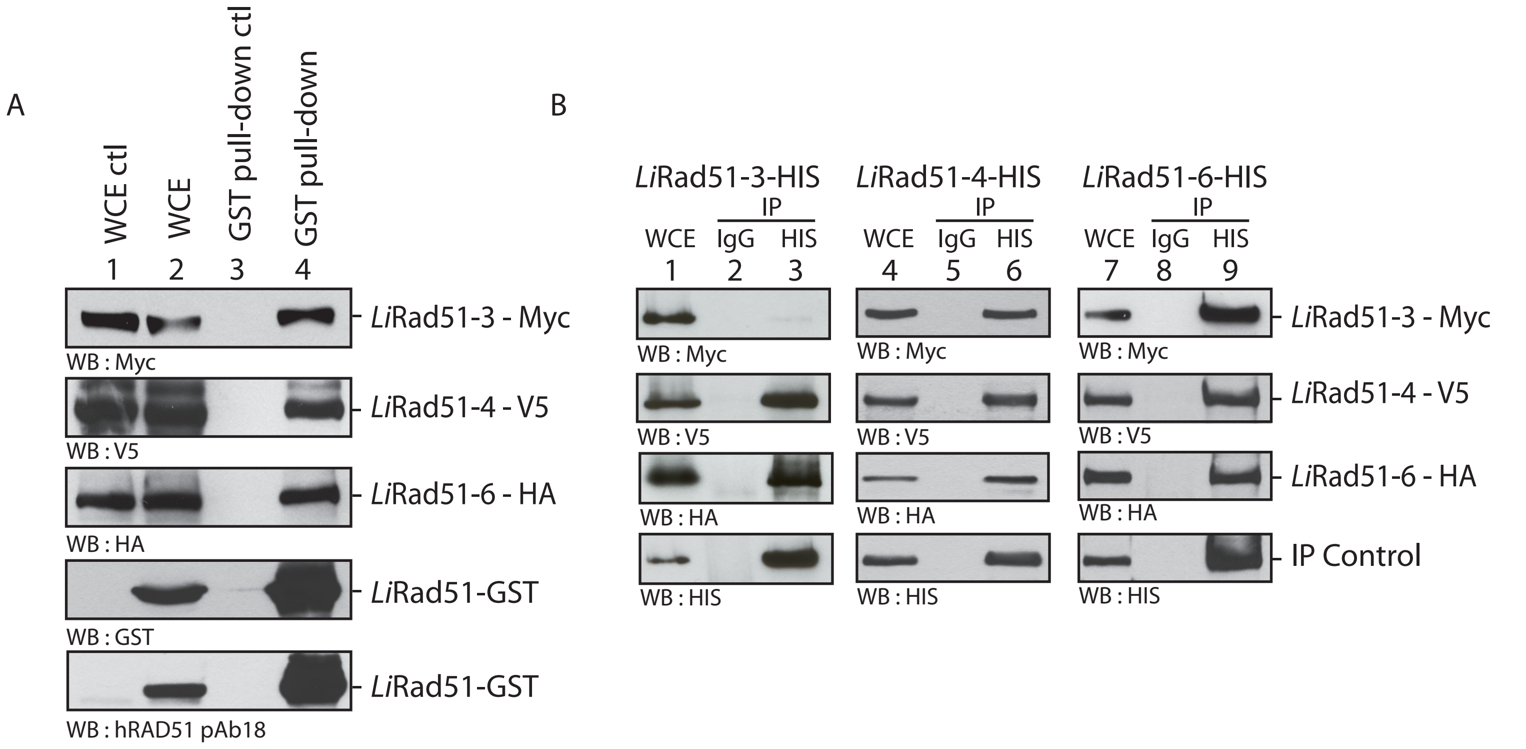

Supplementary Figure S1. (A) *Li*Rad51 interacts with *Li*Rad51 paralogs in Sf9 cells. Insect cells were co-infected with *Li*Rad51- GST and *Li*Rad51-3 MYC or *Li*Rad51-4 V5 or *Li*Rad51-6 HA baculoviruses, followed by GST pull-down experiment. Blots were reacted with the indicated antibodies. Lanes 1-2, whole cell extract; lanes 3-4, GST pull-down. As controls, cells in lane in 1 and 3 were not infected with the *Li*Rad51-GST baculovirus.
(B) Self-interaction of *Li*Rad51 paralogs. Insect cells were co-infected with either *Li*Rad51-3 HIS (lane 1-3) or *Li*Rad51-4 HIS (lane 4-6) or *Li*Rad51-6 HIS (lane 7-9) with *Li*Rad51-3 MYC or *Li*Rad51-4 V5 or *Li*Rad51-6 HA baculoviruses. Immunoprecipitations using an anti-His antibody were conducted and blots were reacted with the indicated antibodies. Lanes 1,4,7, whole cell extract; lanes 2,5,8, immunoprecipitations with IgG alone; lanes 3,6,9 immunoprecipitations with anti-His.

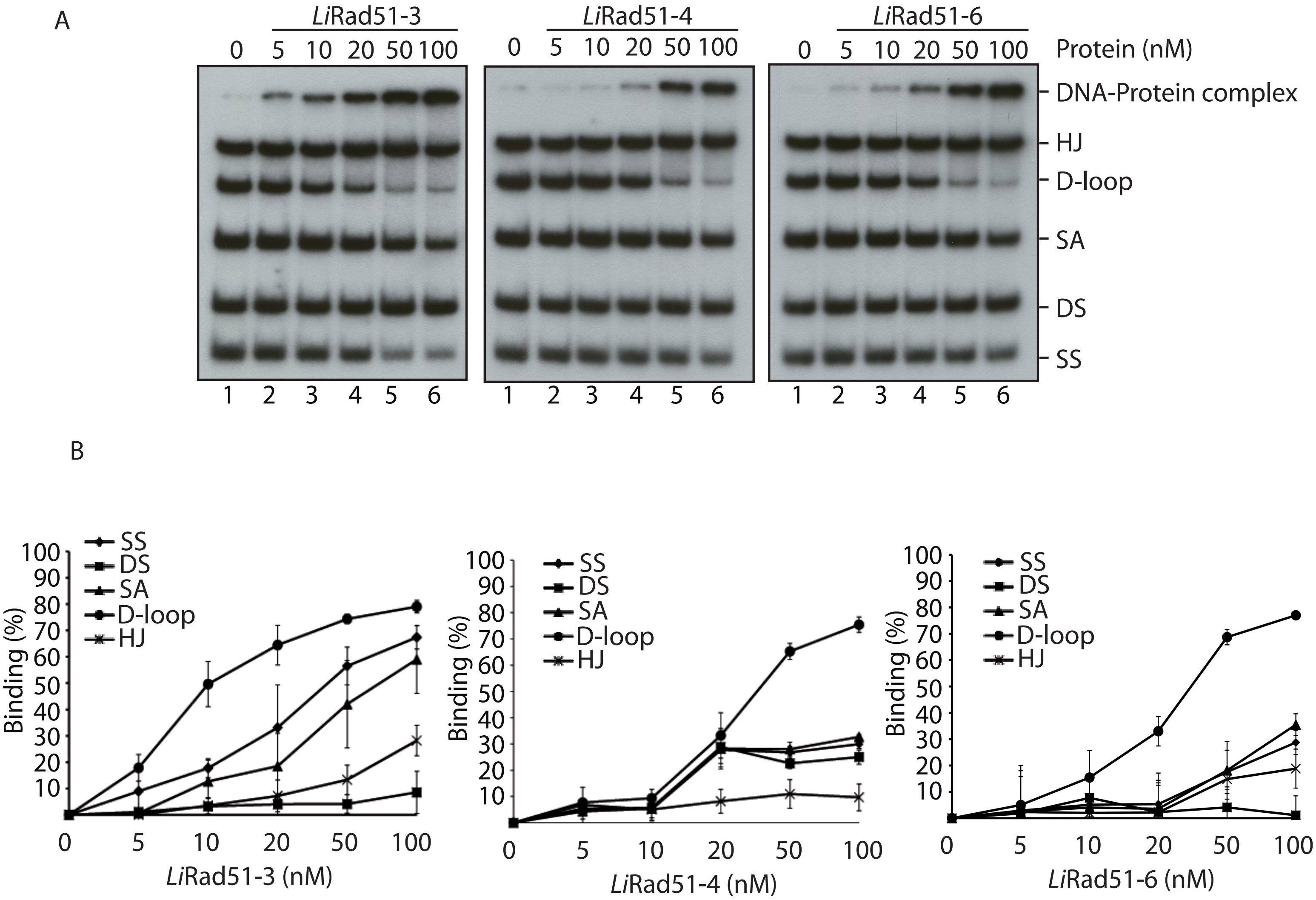

Supplementary Figure S2. *Li*Rad51 paralogs bind preferentially a D-loop substrate.
(A) Competition EMSA were performed with increasing concentration (0-100 nM, lane 1-6) of *Li*Rad51-3, *Li*Rad51-4 or *Li*Rad51-6 with Holliday junctions (HJ), D-loop, splayed arm (SA), dsDNA and ssDNA. Complexes were migrated on 8 % acrylamide gel.
(B) Quantification of DNA binding where error bars represent SD from three independent experiments.

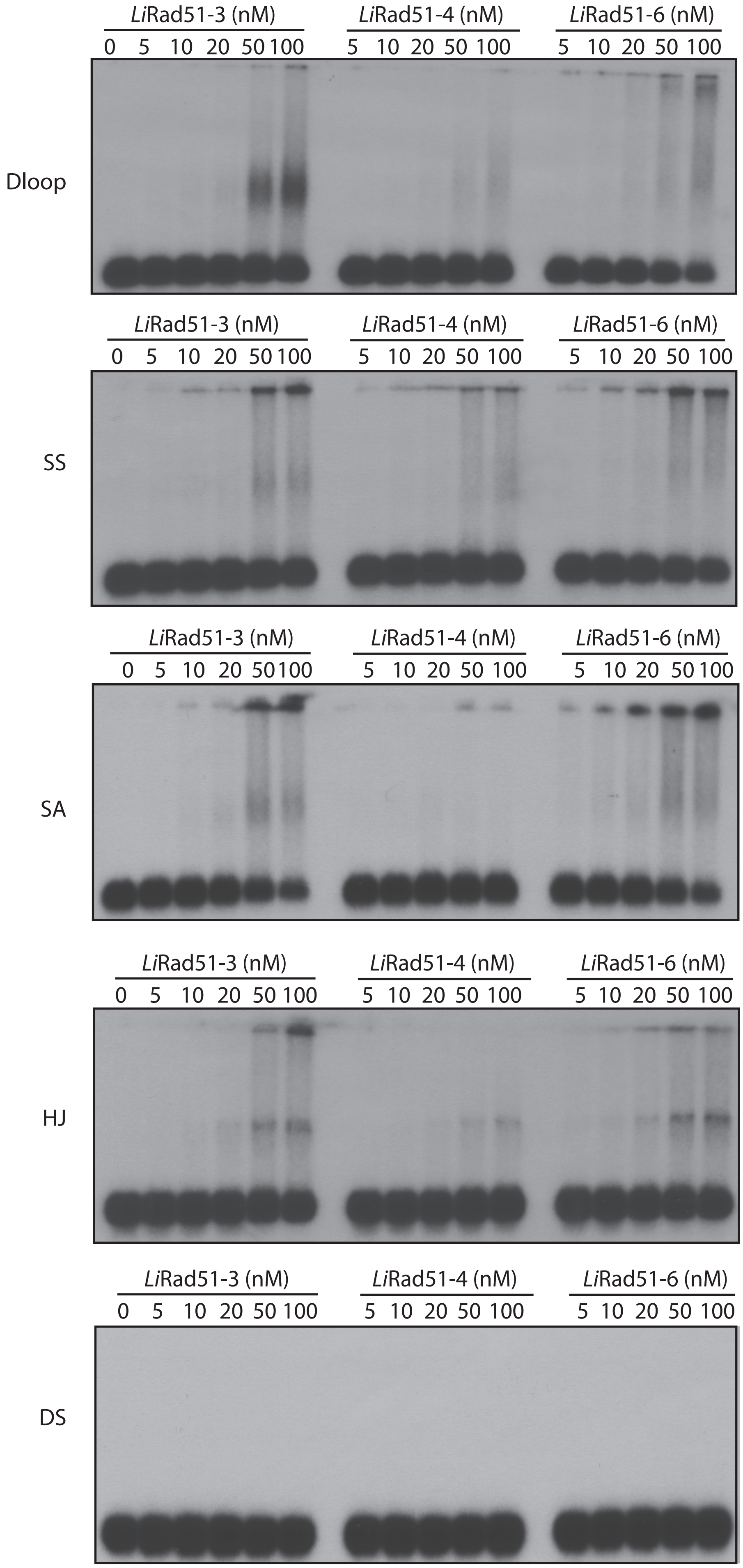

Supplementary Figure S3. DNA binding of each paralog (0-100 nM) with 5′-labelled probes. Complexes were migrated on agarose gel (0.8%).

A

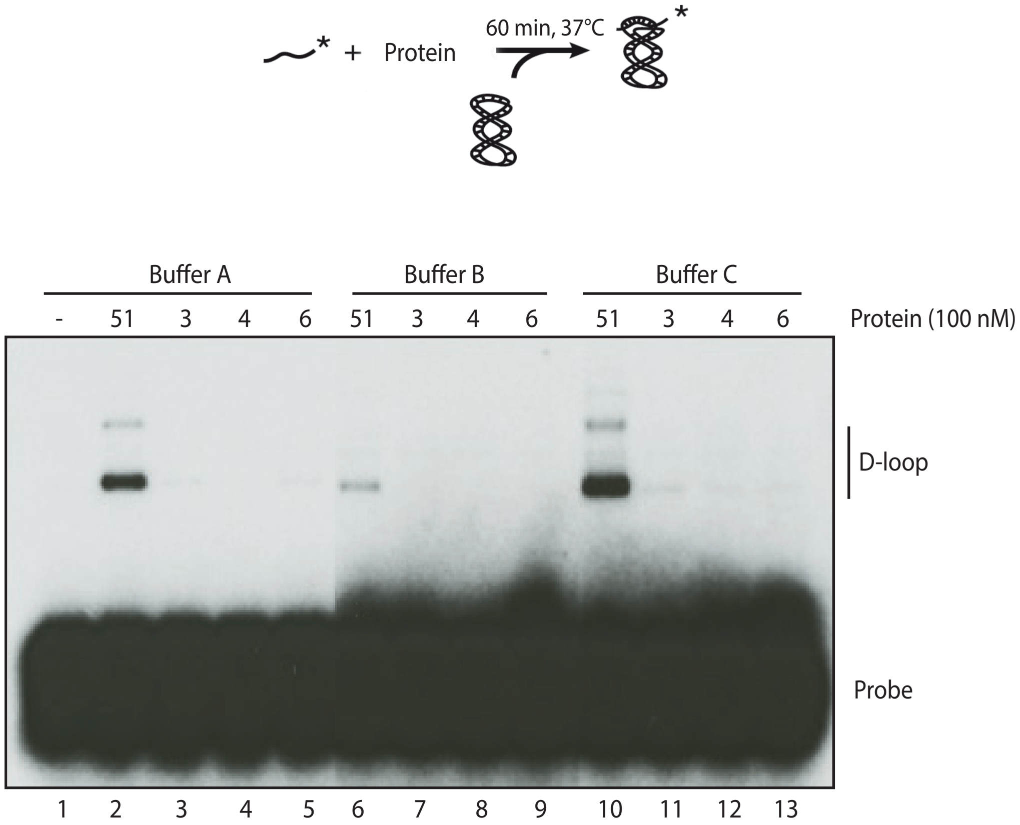

B

Buffer A

25 mM Tris Ac pH 7,5
2 mM CaCl2
2 mM ATP
1 mM DTT
20 mM creatine phosphate
5U/mL phosphocreatine kinase

Ref : This study

Buffer B

25 mM Tris Ac pH 7,5
2 mM CaCl2
1 mM ATP
1 mM MgAc
2 mM DTT
100 µg/mL BSA
20 mM creatine phosphate
30U/mL phosphocreatine kinase

Ref : Bugreev *et al.* Gene Dev., 2007

Buffer C

25 mM Tris Ac pH 7,5
5 mM CaCl2
2 mM MgCl2
2 mM ATP
1 mM DTT
100 µg/mL BSA

Ref : Barber *et al.* Cell, 2008

Supplementary Figure S4. (A) Top: Schematic representation of D-loop assay using a labeled 100-mer single-strand DNA and supercoiled DNA template. Bottom: *Li*Rad51, *Li*Rad51-3, *Li*Rad51-4 and *Li*Rad51-6 strand invasion on their own in three different buffers. *Li*Rad51 is used as a positive control. (B) Composition of each buffer used.

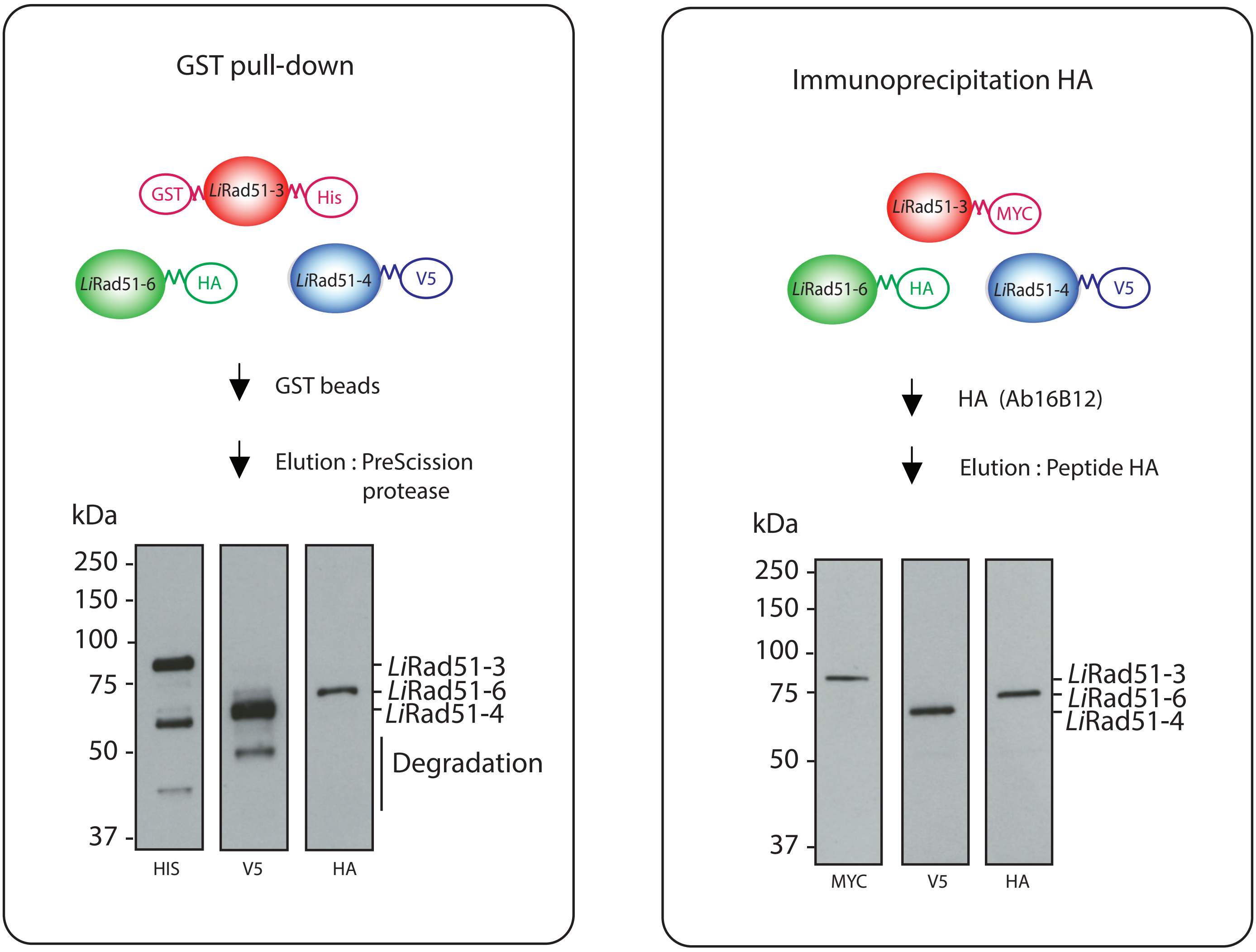

Supplementary Figure S5. A LiRad51 3-4-6 co-complex is present through GST pull-down of LiRad51-3 and PreScission elution or immunoprecipitation of LiRad51-6 using anti-HA antibody.

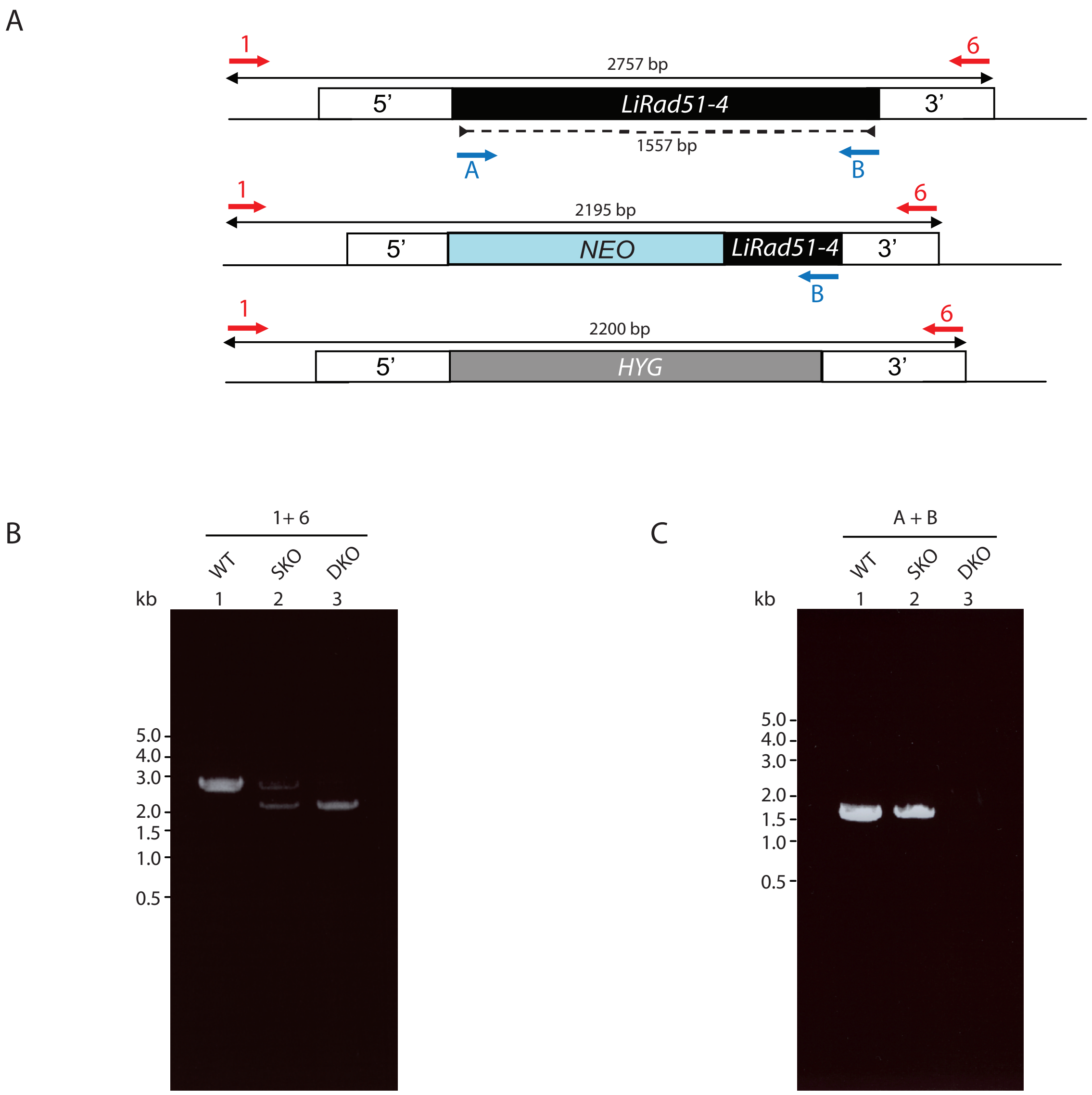

Supplementary Figure S6. Deletion of *LiRad51-4* segment by PCR analysis. (A) Schematic representation of the primers used (red and blue arrows). (B) PCR showing the right integration of the markers in the locus of *LiRad51-4* and the loss of the gene by using primers located in 5′ and 3′ UTR regions (primers 1-6).
(C) PCR showing the complete deletion of the gene by using primers at the beginning and the end of the gene (primers A-B).

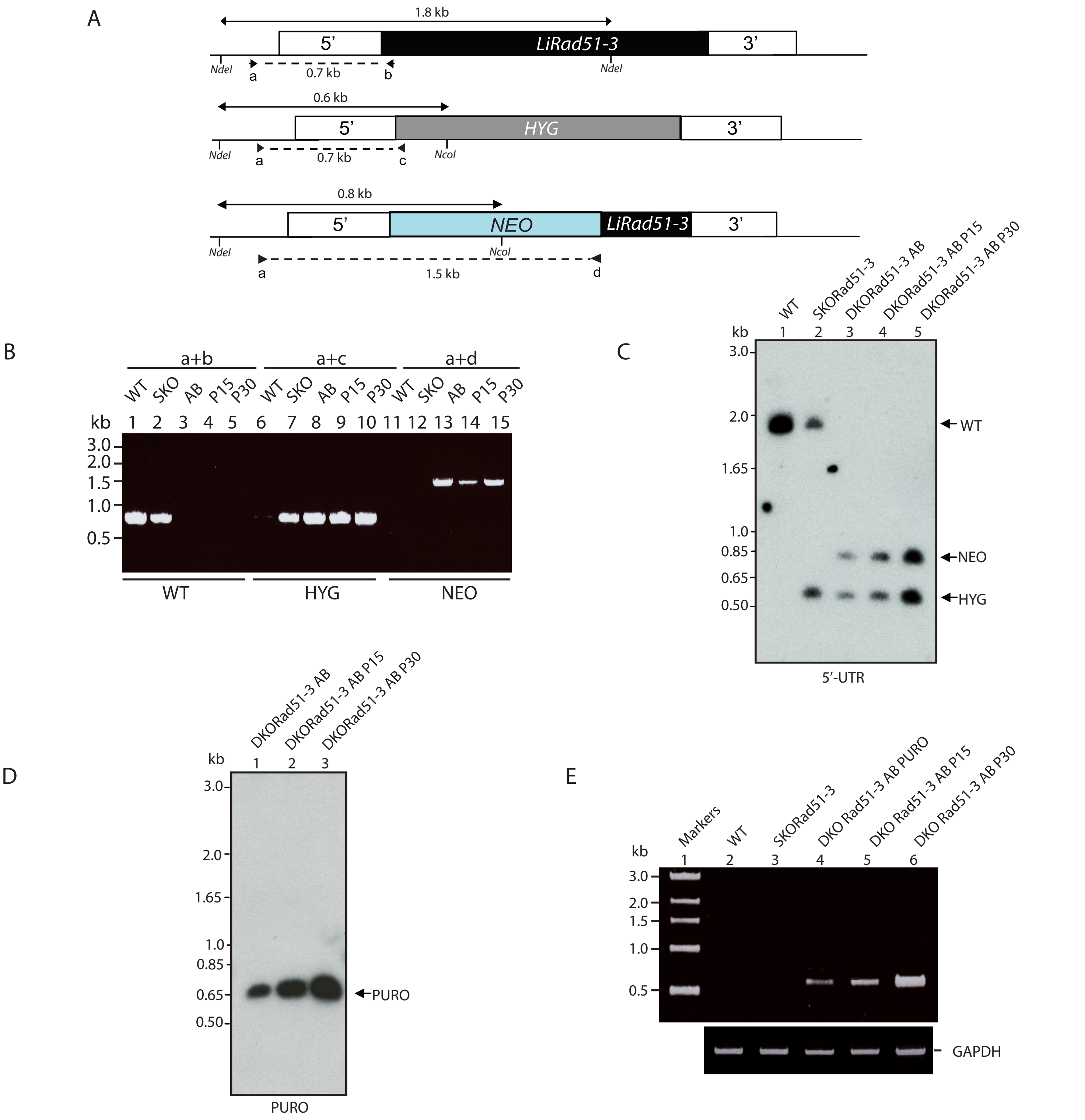

Supplementary Figure S7. Inactivation of *LiRad51-3*. (A) Schematic representation of the *Li*Rad51-3 gene (top) and inactivation cassettes with hygromycin phosphotransferase (HYG, middle) and the neomycin phosphotransferase (NEO, bottom). The position of the primers pairs (a+b, a+c, a+d) used in (B) are depicted with arrows. (B) PCR analysis of five different populations after integration of the HYG and NEO cassettes. DNA in lanes 1,6,11 have no integration referred as *L.infantum* wild-type; lanes 2,7,12 have a HYG insertion in one allele; lanes 3,8,13 have both NEO/HYG insertions complemented with a plasmid expressing LiRad51-3 protein, referred as add-back (AB). Lanes 4,9,14 contain DNA from AB after 15 passages without drug selection (puromycin) for the plasmid; while lanes 5,10,15 are after 30 passages. (C) Southern blot of five different populations digested with NdeI and NcoI after hybridizing with 500 bp 5'UTR probe from the cells described in (B). (D) Southern blot of three different populations digested with NdeI et NcoI after hybridizing with PURO probe from the cells described in (B). (E) PCR analysis of five different populations after integration of the HYG and NEO cassettes with or without drug selection for the complemented plasmid by using specific primers to amplify puromycin gene. Amplification of the chromosomal GAPDH gene was used as loading control.
